# Supplementary figures and images for: Sex differences in the association between systemic oxidative stress status and optic nerve head blood flow in normal-tension glaucoma
Source: PLoS One. 2023 Feb 24;18(2):e0282047. doi: 10.1371/journal.pone.0282047 (PMC9955941; doi:10.1371/journal.pone.0282047)

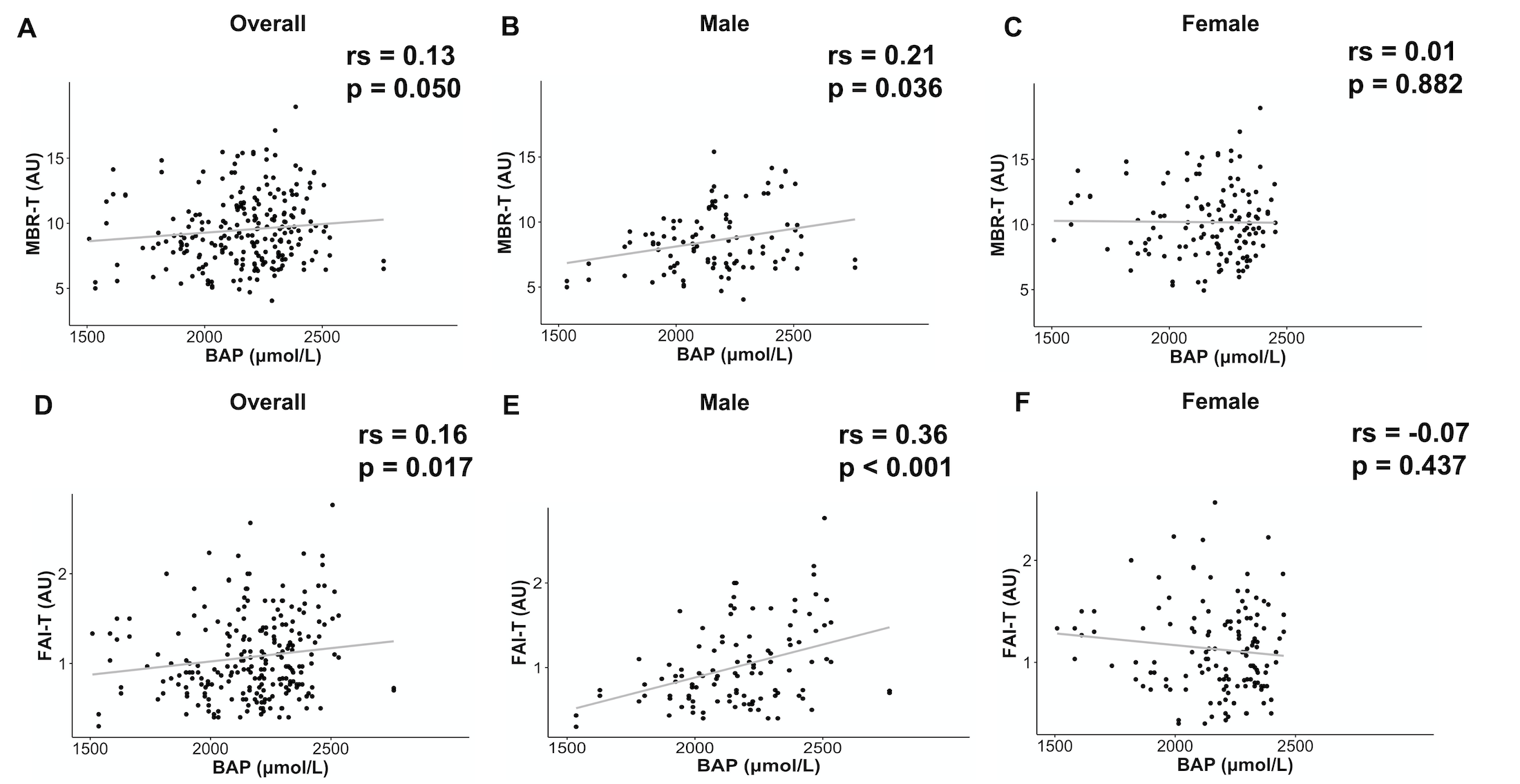

Supplement: S1 Fig — MBR-T was significantly correlated with BAP only in the male patients (rs = 0.21, p = 0.036). FAI-T was significantly correlated with BAP in the overall group (rs = 0.16, p = 0.017) and the male patients (rs = 0.36, p < 0.036). (TIF) [file pone.0282047.s001.tif]
